# A qualitative exploration of prescription opioid injection among street-based drug users in Toronto: behaviours, preferences and drug availability

**DOI:** 10.1186/1477-7517-5-30

**Published:** 2008-10-17

**Authors:** Michelle Firestone, Benedikt Fischer

**Affiliations:** 1 Centre for Addiction and Mental Health (CAMH), Toronto, Canada; 2 University of Toronto, Toronto, Canada; 3 Centre for Applied Research in Mental Health and Addictions (CARMHA), Faculty of Health Sciences, Simon Fraser University, Canada

## Abstract

**Background:**

There is evidence of a high prevalence of prescription opioid (PO) and crack use among street drug users in Toronto. The purpose of this qualitative study was to describe drug use behaviours and preferences as well as the social and environmental context surrounding the use of these drugs among young and old street-based drug injection drug users (IDUs).

**Methods:**

In-depth interviews were conducted with 25 PO injectors. Topics covered included drug use history, types of drugs used, how drugs were purchased and transitions to PO use. Interviews were taped and transcribed. Content analysis was conducted to identify themes.

**Results:**

Five prominent themes emerged from the interviews: 1) Combination of crack and prescription opioids, 2) First injection experience and transition to prescription opioids, 3) Drug preferences and availability, 4) Housing and income and 5) Obtaining drugs. There was consensus that OxyContin and crack were the most commonly available drugs on the streets of Toronto. Drug use preferences and behaviours were influenced by the availability of drugs, the desired effect, ease of administration and expectations around the purity of the drugs. Distinct experiences were observed among younger users as compared to older users. In particular, the initiation of injection drug use and experimentation with POs among younger users was influenced by their experiences on the street, their peers and general curiosity.

**Conclusion:**

Given the current profile of street-based drug market in Toronto and the emergence of crack and POs as two predominant illicit drug groups, understanding drug use patterns and socio-economic factors among younger and older users in this population has important implications for preventive and therapeutic interventions.

## Background

Street drug use is becoming increasingly diversified, both in terms of the types of drugs being used and in the ways in which they are being administered. In Toronto, as in other North American cities, illegal drug markets have been shown to be undergoing a distinct evolution, as prescription opioid (PO) analgesics such as OxyContin, Morphine and Dilaudid are being diverted from medical sources and becoming more widely available to street drug users [1,2]. Simultaneously, recent studies have demonstrated a high prevalence of crack use among urban drug user populations across Canada [3,4]. As a result, in Toronto specifically, there is a clear emergence of two predominant drug groups in the street-based drug market: POs and crack cocaine. The objective of this qualitative study was to gain a clearer understanding of this phenomenon, the associated drug use behaviours and preferences as well as the environmental and social context in which these drugs are obtained and administered among street drug users.

In Canada, it has been estimated that there are between 90,000 and 125,000 injection drug users (IDUs), the vast majority of which are believed to use illicit opioids [5,6]. In North America, increasing prevalence rates of PO misuse have been recently reported for general populations [2,7,8]. According to International Narcotics Control Board (INCB) data, the United States is by far the world's largest consumer of POs on a per capita basis (37,565 defined daily doses of opioid per million population, 2004–06). While Canada ranks as the 4th highest overall consumer (16,628), it is the world's top consumer of specific opioids (e.g., hydromorphone) [9]. Although limited, recent studies have begun to characterize the use of POs among more marginalized populations. For example, a study of illicit opioid-dependent individuals attending a large Toronto Methadone Maintenance Treatment (MMT) program reported oxycodone (46.6%), codeine (45.5%), morphine (21.3%) and hydromorphone (17.4%) as the most prevalently used PO drugs [10]. Furthermore, data from the OPICAN study, a cohort study of illicit opioid and non-opioid drug users conducted in 7 major Canadian cities (Edmonton (Alberta), Montreal, Quebec City (Quebec), Toronto (Ontario), Vancouver (British Columbia), Fredericton and St. John (New Brunswick)), revealed that heroin use had significantly decreased in the study population between 2001 and 2005, and that PO use was more prevalent than heroin use in 5 of the 7 study sites [11]. Finally, while data on PO abuse among younger populations in Canada is limited, results from the 2007 Ontario Student Drug Use and Health Survey indicated that one in five (21%) students in grades 7 to 12 reported the use of opioid pain relievers for nonmedical use in the past year [12]. Upsurges in OxyContin abuse among youth have also been reported in Eastern Canada, including St. John's, Newfoundland [13].

As with PO abuse, recent studies have demonstrated a high prevalence of crack use in street drug populations across Canada [3,4]. A survey of IDUs in Toronto, Regina, Sudbury, and Victoria revealed that 52.2% of the total sample had used crack (non-injection, i.e., smoking) in the last 6 months; in Toronto specifically (n = 221), more than three quarters (78.7%) of those surveyed had smoked crack [3]. Data from the OPICAN cohort indicated that 54.6% of baseline participants had used crack in the past 30 days and 87.2% of those crack users reported smoking the drug [14].

Drug use among opioid user populations has been increasingly diversified, resulting in 'poly-drug use' profiles, which often includes combinations of more than one opioid, but also involves the combining of opioids with other drugs, namely, crack cocaine, benzodiazepines and alcohol. In a Toronto study conducted with regular opiate users not in treatment, 70% had used alcohol, 64% had used cannabis, 61% had used benzodiazepines and 58% had used cocaine [15]. A latent class analysis of the OPICAN data revealed that the cohort participants could be divided into three distinctly different 'drug user type' groups characterized primarily by: heroin and cocaine injection use, other opioid and benzodiazepine use and non-injected other opioid and crack use [16]. Research has also shown that poly-drug use can be linked to distinct morbidity and mortality consequences as well certain socio-demographic characteristics. For example, the co-use of opioids with stimulants (e.g., cocaine, benzodiazepines or alcohol) considerably elevates the risk for fatal and non-fatal overdose [17,18]. Furthermore, in the OPICAN study it was found that co-users of opioids and crack were significantly more likely to be characterized by unstable housing, criminal activity, health problems and injection risks compared to non-co-users of crack [4].

In addition to poly-substance use, the diversification of drug use profiles may also lead to shifts in drug use 'careers' and changes in drug use behaviours, chiefly, the transition from non-injection to injection. The transition dynamics from non-injection to injection drug use has been documented in the literature, particularly within the context of heroin use [19-21]. Factors associated with transitioning from non-injecting to injecting have included demographic characteristics such as male gender and older age [22,23] and economic factors such as employment and housing [24]. In a study conducted with 19 young drug users who had recently transitioned to injection (past 3 years), Sherman et al., found that the social impact of family, friends and sexual partners and the high concentration of injection in their local neighbourhoods were important factors in this shift in drug use behaviour [25]. The influence of a sexual partner who injects drugs has also been linked specifically to the first injection experience [26,27]. Environmental factors and market characteristics can also impact decisions around the route of drug administration. In Spain, de la Fuente et al. observed a strong relationship between heroin purity and route of administration, such that in areas where brown heroin was more prevalent, there was an increased proportion of chasers ('chasing the dragon' or inhaling the smoke from heroin) as compared to areas where white heroin and resulting intravenous use were more prevalent [28]. Finally, compared to non-injectors, IDUs are generally in poorer physical and mental health as compared to non-injectors and are substantially more likely to have been infected with HIV, Hepatitis B (HBV) or Hepatitis C (HCV) [29-32].

There are two predominant groups of illicit drugs emerging in the street drug use setting of Toronto, namely crack cocaine and POs. In this qualitative study, we aim to examine the driving forces behind the combinations of different types of drugs, the context and environment in which these drugs are obtained and administered, and their availability and appeal among a small sample of street-based IDUs.

## Methods

Between March and June 2007, 25 PO injectors who had injected at least once in the previous month and who had used crack in the past 6 months were recruited in Toronto, Canada. A total of 11 in-depth interviews were conducted with young injectors (18–24 years old), while the remaining 14 interviews were conducted with older injectors (24–50 years old).

Using targeted sampling methods [33], community outreach contacts in three downtown locations informed potential participants about the study. After prospective participants were screened for eligibility and informed consent was obtained, one interviewer conducted all 25 interviews in a private, neutral location at community locales. Interviews were open-ended and exploratory in nature on the basis of a semi-structured interview guide, covering different topic areas such as drug use history, types of drugs used, where and how drugs were purchased, experiences surrounding first injection, transitions to PO use and experiences around addiction treatment and harm reduction programs. Each tape-recorded interview lasted approximately 45 minutes for which participants received $20 as compensation for their time. No identifiers were recorded. After transcribing the interviews, content analysis [34] of the transcripts was conducted to identify the major themes with respect to the topic areas covered by the interview guide. Several main themes were then identified and transcripts were hand-coded in an iterative process whereby codes were reviewed and adjusted in relation to previous codes in order to facilitate a richer understanding of the data. The most salient themes were then organized into matrices and meaningful quotes were extracted from the transcripts. The study protocol was approved by the Research Ethics Board of the Centre for Addiction and Mental Health (CAMH).

## Results

A total of 10 females and 15 males ranging in age from 18 to 50 years old (mean age of 33 years) participated in the study. The mean number of years using any drug was 21 years among the older users (ranged from 12 years to 36 years) and 7 years among the younger users (ranged from 2 years to 11 years). Initiation of PO injection occurred as recently as 6 months and as far back as 10 years prior to the interview. The frequency of PO injection ranged from twice a week to up to 10 times daily, with an average of between 4 and 5 injections a day. In addition to alcohol and marijuana, participants reported current use of non-opioids such as crack, cocaine, Ritalin and Ketamine. Opioids currently being administered included: heroin, OxyContin, Dilaudid, Morphine, Hydromorphone, Talwin, Fentanyl and Percocets.

Five prominent themes emerged from the interviews: 1) Transition and co-use of opioids and crack, 2) Social networks and experiences around first injection 3) Drug preferences and availability, 4) Housing and income generation and 5) Context of obtaining illicit drugs (namely POs and crack). Within these themes, very distinct experiences were observed between the younger and older group of injectors.

### Combination of crack and prescription opioids

Many participants in both the older and younger group of injectors reported recent use of crack (mostly smoking, but a few had also injected). The timing and context of combining crack with POs differed among participants; however, more people expressed a preference for injection of an opioid first, followed by the use of crack than in the reverse order.

'When I wake up in the morning, because I'm sick, the first thing I'll do, I'll use [inject an opioid] first. Once, I've used, if it's early enough for me to somehow get a toke [smoke crack] somewhere and then go out and get a shot afterwards, then, yeah, I'll do that, if not, I'll just work on getting a few shots and then I know I'll be fine and then whatever happens happens.' (Male, age 21)

'No, I have to take the pill first, when I wake up, cuz you're sick, right. But, you'll find when you get your cheque, you'll say, okay, I'm gonna go buy 20 Oxys and half an 8-ball [crack], by the time you're done the 8-ball, you do a few tokes and you wanna come down, so, you use the pill to come down which you know, burns them up real quick. Like, I like to do a 20-piece and have an Oxy afterwards, I do that and then I calm down and then I go back then.' (Male, age 41)

For others, the order, type of drug combination and route of administration depended on a range of environmental and individual factors, which included: drug availability and price, cash flow, proximity to dealers, rotation of veins or collapsed veins and a desire or curiosity for a new and different kind of 'high'.

Younger injectors spoke a lot about the overwhelming and often unavoidable presence of crack on the streets. In some circumstances, the younger population would prefer to use other drugs, but when these could not be located as easily, the temptation and significant quantity of crack on the streets would become too strong. As several young users explained:

I: What about crack?

S: Crack. It's everywhere. Yeah there's so much of it. Sometimes when I want to buy a joint I can't even find it but I can always find crack. (Female, age 19)

'Crack, cocaine and stuff like that, the only reason I started doing that again is because I was back in Toronto. That shit's everywhere. It really is. It's bad.' (Male, age 21)

### First injection experience and transition to prescription opioids

There were distinct differences in the experiences and context of the study populations' first injection of an illicit drug. For about half of the older population, these experiences occurred during their early adolescence and were strongly influenced by family members and friends who were using illicit drugs.

'I started using drugs when I was 13. Heroin. From 9 years old I was hitting them [older family members], playing doctor, they were too fucked up to do it themselves. By 13 I was real curious, watching them get all high, so I was curious. Curiosity got me.' (Female, age 41)

Younger users attributed their initiation of opioid injection to exposure on the street, word of mouth and general curiosity. All of the younger users described experimentation with drugs during high school, which included marijuana, alcohol, ecstasy and Ketamine. A few of the younger participants took POs from family members' medicine cabinets, but the majority were exposed to these drugs through the social networks and experiences of living on the streets.

**I: **Why did you start injecting?

**S: **It was basically curiosity. The people I was hanging out with were doing it and I just tried it. (Female, age 19)

'Yes I was curious about it. Yeah it was offered. Let's just say that the person that got me started me on it was lonely. She needed another junkie buddy. So she basically turned me onto heroin I think for her own personal reasons. One she wanted a friend, you know someone to relate to. Second she needed help getting the money for it.' (Female, age 23)

All participants had a history of drug use prior to using POs. Only one of the younger participants had ever received a prescription for an opioid drug, but she had been using heroin for some time prior to this. While several of the older injectors had obtained POs through legitimate prescriptions following pain problems related to an accident or another illness, for only 3 did this mark their initiation into opioid abuse.

'I got into the opiates. I started off with the percs [Percocets] after my accident and then one thing led to another and I met this massage therapist and I tried a couple lines of heroin and then, wow this is a lot better than cocaine, and I had two jobs, and I started snorting it and then in the last few weeks I started injecting.' (Male, age 41)

'I was in a car accident. I went through the windshield. So, they prescribed me 2 40's [OxyContin] a day, for three consecutive weeks. It didn't take long before I was like, I love these now, I can't cope...' (Female, age 49)

### Drug preference and availability

The overwhelming majority of participants agreed that OxyContin was the most common and readily available PO on the streets of Toronto. Preference for a specific pill varied among individuals and was influenced not only by the availability of the drug, but also depended on the length of high generated and the ease with which certain pills could be broken down, heated and injected as compared to others. In the following quotes, respondents described how they weighed the pros and cons of using different types of POs:

'Well, the morphine is better because it has longer life, but the Oxys are quicker, they're faster and they're easier and a lot of time you're in a public washroom or somewhere, like over there, and then someone banging on the door and you miss half of it. Plus, the Oxys are easier on your system, they don't gel up, like sometimes after the morphine goes in it gels up, it leaves lots of bumps...' (Male, age 41)

'A different buzz. Because when you inject morphine, you get pins and needles. I hate that feeling. From the bottom of your feet to the top of your head. Some people like that, but I don't. And, but, morphine has long legs. It lasts longer. Dilaudids are cleaner, but they don't last as long. You get a nice rush. A cleaner high. You don't get the pins and needles, but it's shorter than the Oxys.' (Female, age 49)

As many participants explained, compared to the poor quality and high price of heroin in the city, POs are appealing and sought after because they are dependable and consistently generate the same psychoactive experiences.

'Yeah, because heroin you never know right? And it's a pharmaceutical process, so it's close to fucking being the same in every pill as humanly possible. It's not like ecstasy where every one is a little bit different. Even these pharmaceutical pills, they can't make them exactly the same, but it's pretty close.' (Male, age 21)

**I: **So, how did you make the switch from heroin to the morphine?

**S: **Availability...and I got some bad dope. I was having seizures. So, pharmaceuticals I always know what I'm getting all the time. (Male, age 38)

Overall, people described their preference for the opioids over other drugs like crack, due to their calming effect, the numbness they experience and the overall feeling of normalcy produced.

'Oxys are opposite of cocaine. Mellow. Makes me normal. Without it I can't get out of bed. Now it's just to make me get up and walk and go. To do my daily things. Like a heroin addict. Just to go to work and do things.' (Female, age 49)

'Cause I don't like being sketched out all the time and being awake for fucking between 3 days and a week or how ever long you are awake. And feeling like shit. Opiates don't really do that. Opiates are still, you know, you're the exact same person, just maybe with a better sense of wellbeing. A little more euphoria than normal. But you don't sketch out at all. It's just a lot harder on the body. And the uppers, the uppers just fuck with your brain...in a lot of ways.' (Male, age 23)

### Housing and income

Both older and younger opioid injectors described unstable housing and unreliable income sources. Only 3 of the older participants reported that they were currently living in their own place. The remaining interviewees currently resided at family or friends' homes, stayed in shelters or were living on the streets. Interestingly, more of the older injectors relied on the shelter system in the city, while several of the younger participants described their dislike and avoidance of the shelters due to the curfews and rules in place and a lack of cleanliness, security and privacy.

I: You don't like the shelters?

S: It's like living with my fucking parents all over again. It's not only that-it's the people. I've been ripped off so many times in the shelters. I've been robbed more in the shelters than I ever have on the streets. I'm safer on the streets. In most cases. There's bugs and creeps. (Male, age 21)

While none of the participants were asked directly about their sources of income, many did disclose information about how they were supporting their drug habit. Two of the female participants, for example, did refer to their involvement in sex work; however the vast majority of this population earned money by panhandling and relied heavily upon monthly social assistance from the government (i.e. welfare, disability). Among all of the participants, there was consensus that supporting their use of POs and crack was a very costly endeavour.

'I do about 4 or 5 80's a day [80 mg of OxyContin]. That's $125 a day. It adds up. I have to work. I work the street for that. It's a drag. I used to have a full time job, but now I have to, I can't wait two weeks for my pay.' (Female, age 49)

'Actually, say like $75, probably $60 bucks is on pills. Well like on heavier days I've spent like closer to like $200, but that would be like an average day, probably like a dime of pot a beer and like 4 Dilaudids or 2 Oxys.' (Male, age 23)

### Obtaining drugs

The vast majority of participants currently purchased POs and crack from various dealers throughout the city. Both young and older injectors described how specific dealers sell specific drugs. While one dealer may get more than one PO at a time, this same dealer would not likely sell crack. The distinction between these dealers would go beyond the drugs they sell, but also included their trustworthiness, their visibility on the streets and their selectiveness in whom they sell to.

'It's harder to get the opiates than the crack. Crack you can find on any corner. Opiate dealers are old school. They're getting it from their doctors.' (Female, age 41)

'Nope, no they're not [pills are not easy to find]. And a lot of opiate dealers are careful of the longer jail times [for selling]. And they're more picky with who they deal with. Very picky. Whereas the crack users are like, "Come, everybody in the alley!"' (Female, age 23)

The older participants had a better understanding of how their dealer was acquiring the POs, as they seemed to have established closer relationships with their dealers and loyalty towards them as compared to the younger injectors who expressed more uncertainty and even reluctance to inquire about sources of drugs. Given the short length of their injection careers, several younger users explained how they were first introduced to dealers by other PO injectors and how they had to establish trust over time.

'Like a friend of mine gets morphine, he gets 100 greys [80 mg tablets of prescribed morphine] every month and he's never even, he's eaten a half of one just so he had it in his system when he went in for his hip operation, but as long as they don't start taking them, then they don't get addicted to them and then they sell them each for $10 bucks and then they turn around and buy crack.' (Male, age 41)

**I: **Since you are a pretty new user did you have to get to know the right people?

**S: **Yeah. Now I know dealers that I just go see myself, but when I first started I had to get other users to go find pills for me.

**I: **Did you have to give them a cut?

**S: **Yeah, usually you have to give them 5 bucks for making the run, you know, or give them a piece of the pill or something. (Male, age 20)

**I: **Do you know where the dealer gets them?

**S: **No idea. No clue. I don't really want to ask them.

**I: **Did someone else have to hook you up?

**S: **Yeah, someone else introduced me to and said, this guy is cool. (Male, age 19)

Younger participants also described trading drugs like marijuana for pills or crack or the residue ('wash') in their spoon for a toke of crack and therefore would obtain drugs through less formal interactions.

**I: **Is there a lot of trading?

**S: **People like weed around here. If you have weed, you can trade for crack, the pills or ecstasy. (Male, age 24)

'On average, it's hard to say, I don't have that much money, but I'm panning $200/week on pills and stuff. I usually get pot and stuff for free. It's just the pills I pay for. Crack I usually get offered for weed. I just trade for some weed.' (Male, age 24)

## Discussion

Results from this qualitative study point to the specific drug preferences, combinations, and circumstances surrounding initiation, availability and acquisition of POs among a small sample of street-based drug users in Toronto. Within this context, distinct experiences were observed between the older and younger participants whose exposure to and experimentation with POs occurred at a very different stage of their drug use 'career'.

According to study participants, decision making around the types of POs used, frequency of use and quantity administered appeared to be influenced by a combination of factors. Unanimously, participants agreed that OxyContin is the most easily procured PO in Toronto. Similar trends have been observed in Toronto among opioid users enrolled in MMT who reported oxycodone use more frequently than other POs and among street drug users in other North American cities [10,13,35]. For both the older and younger participants, the overwhelming majority of drugs purchased or traded occurred by way of a dealer. There were speculations that dealers either possessed a legitimate prescription or were obtaining drugs from a third party with a prescription, or that drugs were acquired through pharmacy thefts and from raids of expired prescriptions being removed from private homes or medical institutions. Similar findings were observed in a study by Inciardi et al., in which drug abusers in Miami, Florida cited a range of sources of prescription drug diversion including their physicians and pharmacists; parents and relatives; "doctor shopping"; leftover supplies; direct sales on the street and in nightclubs; pharmacy and hospital theft; flyers and advertisements etc. [36].

The implications for prevention, particularly in Canada, point to more consistently enforced prescription drug control measures across the provinces. In response to growing concerns about the prescribing and usage of controlled substances, particularly oxycodone products in Atlantic Canada, the government has consulted with key stakeholders and licensing authorities for pharmacists and physicians and issued a report in 2005 on the retail sales transactions of oxycodone-based products in this region [37]. However, additional research focused on the social networks and avenues through which drugs are obtained and sold and involving the individuals with direct sources and access to POs is needed.

When asked which drugs were preferred if finances and availability were not obstacles, choices appeared to be influenced by the desired effects, ease of administration and expectations around the purity of the drugs. So, for example, morphine might be favoured for its 'longer legs' (length of high) and the 'pins and needles' sensation it causes, despite difficulties with preparation. Alternatively, some study participants associated OxyContin and Dilaudid with a cleaner and quicker preparation method, which was considered desirable if he or she relied on public spaces, e.g., restrooms, to inject. An additional appeal of the POs, particularly for heroin users who are dissatisfied with its quality in the city, is the consistency of dosage and purity associated with a pharmaceutically manufactured product. All of these logistical issues and preferred behaviours must be considered from a prevention or harm reduction platform, particularly in the context of the realities of street drug use. Exemplary programs such as InSite, the safe injection site in Vancouver emerged in response to the concentration of heroin use in Vancouver's east side and associated high numbers of overdoses [38,39]. In Toronto, the establishment of a safe consumption site is recommended for exploration by the City's Drugs Strategy, yet, to date, has not materialized [40]. The distribution of 'safer crack use kits' is one harm reduction strategy that targets the city's current drug profile [41], however, by further understanding the daily realities of street-based users in Toronto, programming initiatives can be better tailored to meet their needs, perhaps through the provision of harm reduction kits that include information and tools on the safer use of POs.

Poly-substance abuse was highly prevalent among study participants who reported using combinations of different POs as well as combinations of opioids with non-opioid drugs. All but a few of the participants were currently combining their PO use with crack in some form and while some users described using opioids to come down from crack, the combination of drugs was not always so deliberate or planned, particularly because the ease of acquiring crack far exceeded that of pills. In fact, several of the younger participants expressed desire to reduce or eliminate their use of crack, but given its ubiquitous presence on the streets of Toronto and how frequently it is traded or shared without the need for payment, it is often very difficult to avoid the temptation. The high prevalence of crack use in Toronto and the health and social impact of the drug from the users' perspective has been documented previously [3,42,43]. Given that treatment options specifically for crack dependence are limited in both scope and effectiveness [44,45], prevention and outreach efforts in the form of drop-ins or support networks would offer crack users a safer, more accessible alternative to remaining on the street, surrounded by temptations and would serve as a positive venue for additional harm reduction and information exchange.

Many of the younger participants reported a history of stimulant use (e.g. Ketamine, ecstasy and crack), but were relatively new to opiates and expressed a preference for the calming high of opiates and the sense of normalcy they produce as compared to crack. This population is also quickly discovering that the most intense opiate high is achieved through injecting. The decision to inject marks a crucial turning point in a drug user's 'career' which is of great concern given this populations' inexperience with injection and the increased exposure to the transmission of blood borne diseases, such as HIV, hepatitis C and hepatitis B, drug overdose and other morbidities associated with this practice [29,46-49]. As previously stated, there are many factors influencing shifts in drug careers and only a few studies have explored the possibility of interventions to prevent the transition from non-injecting to injecting [50,51]. The window of opportunity for prevention of disease transmission and reduction of risk exists very early on during an IDUs' career, meaning that prevention efforts and harm reduction messages must be directed towards this younger population who are new to injection [52].

The vast majority of the study participants were currently living in unstable housing or on the streets and financing their drug use through social welfare cheques and panhandling. In many cases, the younger participants avoided shelters and transitional housing and preferred to live on the streets where there were no rules, a greater sense of independence and an existing network of peers to engage with and depend on. Clearly, social network dynamics among younger users had a strong impact on their drug use practices and specifically, the initiation of injection drug use. The influence of friends, sexual partners and family members on patterns of drug use and transitions in drug administration routes has been observed in a number of settings [27,53,54] and among younger users [25]. In Australia, Crofts et al., found in a sample of young IDUs, 65% described their first injection episode as unplanned and only 12% injected themselves the first time [26]. Furthermore, research studies have shown that peer pressure and perceived expectations among IDUs are determinants of risky injection practices [55]. Building on existing social networks and the strong influence of peers within this population would increase breadth and positive impact of existing prevention efforts and outreach services that target street-based youth in urban centers.

Additional environmental factors have also been linked to shifts in drug administration routes, particularly among street-based drug users. For example, in an ethnographic exploration of heroin markets in New York City, Andrade et al. found that changes in the supply of heroin at wholesale and retail levels; the decentralization of the heroin market from dealer-based to user-centred; and declines in heroin quality, prompted non-injection heroin users to develop tactics which may have lead to initiation of injection [53]. Furthermore, the social processes which lead to initiation of crack use in inner-city environments have also been explored in a study by Fagan and Ko-lin who found that initiation of crack use was associated with users' extensive involvement in drug selling and non-drug crimes, changes in drug markets and availability and the decline of economic and neighbourhood cohesion in U.S. inner cities [56]. The results generated from this qualitative study in Toronto highlight the current emergence of crack and POs as two predominant drug groups and reveal some of the individual-level factors associated with this phenomenon. Clearly there are broader, ecological features impacting the drug market, drug use behaviour and associated risks of PO injection and crack use in this setting, which need to be explored further.

The data from this study may be limited given that our sample size is small and recruitment occurred via three outreach/health centres in downtown Toronto. Therefore, it is possible that the experiences of less visible PO users were underrepresented in this study. Nevertheless, we observed saturation on a number of themes, which provides confidence that our findings are meaningful. A larger study would not only validate these results, but would expand upon ideas presented here.

## Conclusion

In recent years, the nonmedical use of opioid analgesics has become a major public health concern. The rich narratives of 25 socially marginalized drug users living in downtown Toronto illuminated our understanding of how POs are obtained in this environment, their availability and appeal and what drives young drug users to initiate PO use and injection. Younger users who may be in the process of transitioning from stimulant and cannabis use to an opioid injection 'career' are at particularly high risk for infection and overdose, all of which is exacerbated by binging and experimentation with drugs and dosage (e.g. Fentanyl), homelessness and at times, un-sterile drug preparation practices. While many agencies in Toronto are dedicated to serving such hard-to-reach populations, by expanding peer-based initiatives that work through existing networks-whether these are based on types of drugs used, income generating activities, or geographical location of 'housing'-as a means to access users who are less visible and to inform the population about the risks of using opioid analgesics in ways they were not intended for, would target their efforts more effectively. It is anticipated that the results from this study will not only stimulate additional research on the context and dynamics of PO abuse among street-based populations in Canada and elsewhere, but also lead to more comprehensive programs and services that address the diverse needs of these populations.

## Competing interests

Both authors declare no financial or non-financial competing interests (political, personal, religious, ideological, academic, intellectual, commercial or any other) in relation to this manuscript.

## Authors' contributions

MF and BF co-developed the study protocol. MF coordinated the field-work, conducted interviews, led the analysis of the interview data, and led the manuscript writing. BF contributed to data interpretation and manuscript writing.
